# Investigating the Relationship between Body Mass Index, Cholesterol, and Cancer in United States Adults: A Cross-Sectional Study

**DOI:** 10.3390/diseases12060120

**Published:** 2024-06-04

**Authors:** Anastasija Martinović, David R. Axon

**Affiliations:** College of Pharmacy, University of Arizona, Tucson, AZ 85721, USA; draxon@arizona.edu

**Keywords:** BMI, cholesterol, cancer, cross-sectional-study, United States adults, logistic regression

## Abstract

The purpose of this cross-sectional study was to investigate the relationship between Body Mass Index (BMI), cholesterol, and cancer in United States (US) adults. Data were collected from the 2020 Medical Expenditure Panel Survey (MEPS). Eligible participants were US adults (≥18 years) with data on BMI, cholesterol, and cancer status, who were alive at the end of the data collection period. An adjusted logistic regression model assessed associations between eight possible combinations of BMI and cholesterol status (independent variable) with cancer diagnosis (dependent variable). Among 27,805 individuals in the 2020 MEPS data, 20,818 met the eligibility criteria (weighted N = 252,340,615). Of these 2668 (weighted N = 29,770,359) had cancer and 18,150 (weighted N = 222,570,256) did not have cancer. In the adjusted logistic regression model, underweight and normal weight individuals with high cholesterol were associated with higher odds of cancer (odds ratio, OR = 2.002, and 95% confidence interval, CI = 1.032–3.885, and OR = 1.326 and 95% CI = 1.047–1.681, respectively), while obese individuals with normal cholesterol were associated with lower odds of cancer (OR = 0.681; 95% CI = 0.543–0.853) compared to normal weight individuals with normal cholesterol. This study offers insights into specific groups of individuals who may be prioritized for cancer prevention. Further research is required to investigate these findings in additional subpopulations.

## 1. Introduction

Cancer is defined as the proliferation of cells that have evaded central endogenous control mechanisms. Cancers are typically grouped according to the organ or tissue of origin as well as the molecular characteristics of the respective cancer cells [1]. The most common cancers worldwide are breast, lung, colon and rectum, and prostate cancers [2,3]. Cancer is the second leading cause of death in the United States (US) [4] after heart disease, with a third of all deaths resulting from cancer due to high Body Mass Index (BMI), tobacco usage, low fruit and vegetable consumption, alcohol usage, and lack of physical activity [2]. In 2020, the total number of new cancer cases was 18,094,716 [5] and there were nearly ten million deaths from cancer globally. The cancer mortality and incidence are projected to increase on a global level [6], further increasing the impact on healthcare systems and communities internationally.

A person’s weight is related to their health status and all-cause mortality [7]. Moreover, being overweight or underweight is a known risk factor for a shifted metabolism which may contribute to the development of severe pathologies [8]. BMI is defined as weight (kg)/height^2^ (m^2^), according to the World Health Organization (WHO). A BMI between 18.5 and 24.9 kg/m^2^ is considered normal weight [9]. BMI is the most commonly used measure of people’s weight, although other measurements, such as waist circumference, may also be used [10]. Recent WHO data from 2022 related to people’s weight shows some concerning numbers—globally, 2.5 billion adults (43%) were overweight, of whom 890 million were obese. Moreover, 37 million children under 5 years and 390 million children between 5 and 19 years were overweight, while 390 million adults were underweight globally [11,12]. These data reflect serious and lasting problems for individuals, families, communities, countries, and their healthcare systems. 

Cholesterol is a lipophilic molecule that has many roles which contribute to normal cell functioning. Cholesterol is essential for human life as a component of cell membranes and a precursor molecule for synthesis of vitamin D and steroids, among many other roles. However, cholesterol can be harmful if it reaches abnormally high blood concentrations [13]. High cholesterol (hypercholesterolemia) is a well-known risk for coronary heart disease and stroke [14,15]. Improving diet and lifestyle is a critical component of the American Heart Association’s (AHA) strategy for cardiovascular disease risk reduction in the general population. High cholesterol is often managed by adjusting these lifestyle factors and taking cholesterol-lowering medicines, such as Statins [16]. However, the role of cholesterol in cancer remains controversial since existing studies indicate contradictory results [17]. There are studies that support the intuitive claim that high cholesterol is related to a risk for certain cancer types [18,19,20], but there are also studies that support there is no relation between cholesterol level and cancer [21,22,23]. Additionally, some studies relate low cholesterol level with cancer [24,25].

Previous research has investigated the association of weight, or BMI, and cholesterol on cancer separately. For instance, high BMI is recognized as an important predictor of cancer risk [26], while excess body weight is attributable to about 7% of all cancer deaths [27]. However, there are no current studies that examine the combined effect of BMI and cholesterol level on cancer. To address this gap in the literature, the purpose of this study was to build mutually exclusive groups for various combinations of BMI and cholesterol and assess their association with overall cancer diagnosis.

## 2. Materials and Methods

The Medical Expenditure Panel Survey (MEPS) is a combination of a plethora of large-scale surveys. It provides data on both individuals and families. Aside from the information on employers and providers, the dataset also encapsulates costs, frequency of use, payment methods, and insurance coverage of healthcare services. MEPS has two major components: Household and Insurance. The household component contains self-reported data about each member of a household, such as demography, health condition, status, and care expenditure. The insurance component is a survey on employer-based health insurance [28]. In this work, we used the 2020 MEPS full-year consolidated data file which provides data for MEPS participants throughout 2020 [29]. The respondents voluntarily participate in the survey and provide appropriate oral informed consent. The University of Arizona Institutional Review Board approved this study (STUDY00004073). Eligible participants for this study were those who had existing data on cholesterol level, BMI, and cancer status, were 18 years of age or older, and were alive during the whole data collection period [30,31].

The dependent variable for this study was cancer status—whether the person had ever been diagnosed as having cancer or a malignancy of any kind [30,31]. The independent variable for this study consisted of eight groups, constructed from binned values of two variables—cholesterol level and BMI. BMI is calculated as weight in kilograms divided by the square of height in meters. BMI is an inexpensive and easy screening method for weight category—classifying the population into underweight (bellow 18.5), healthy weight (18.5–24.9), overweight (25.0–29.9), and obese (30.0 and above) [32]. The cholesterol variable ascertained whether the person had ever been diagnosed as having high cholesterol (yes/no) [30,31]. The eight categories of independent variable for this study were following: obese/high cholesterol (OHC), overweight/high cholesterol (OWHC), underweight/high cholesterol (UWHC), normal weight/high cholesterol (NWHC), obese/normal cholesterol (ONC), overweight/normal cholesterol (OWNC), underweight/normal cholesterol (UWNC), and normal weight/normal cholesterol (NWNC). NWNC was the reference group. Categories were formed following the method used by Cao et al. [33], using cholesterol level instead of metabolic syndrome.

Potential covariates in this study were sex, age, race, ethnicity, education levels, employment and marital status, alcohol and smoking status, diabetes, and cardiovascular disease. Sex considered if the participant was male or female. By age, the sample was divided into two groups—younger than 30 years and 30 years and older. Race and ethnicity questions were asked for each member during the MEPS interview. The race variable consisted of four groups—white, black, Asian/native Hawaiian/pacific islander, and other. Ethnicity noted if the individual was Hispanic or not. Education status considered years of education completed when entering MEPS for individuals five years and older. The cut-off point for this study was set at completed high school and resulted in two groups of individuals—people with high school or less and people who had a higher level of education than high school. Employment status was sampled for all individuals 16 years and older. Possible responses were “currently employed”, “has a job to return to”, “employed during the reference period”, and “not employed with no job to return to”. Positive answers on the first two questions formed one cohort for this study and positive answers on the other two formed the other. Current marital status denoted if the person was married or not. In terms of alcohol consumption, participants were asked the question “How often do you have a drink containing alcohol?” and were grouped for the purpose of this study into three groups—people who never drink alcohol, people who consume one drink or less per week, and people who consume more than one drink per week. Smoking status denoted how often participants smoke cigarettes. From answer choices “every day”, “some days”, and “not at all” two groups of people were formed—people who never smoke and people who smoke sometimes or every day. Diabetes status indicated whether the participant had ever been diagnosed with diabetes or not (excluding gestational diabetes). Cardiovascular disease variable was formed from five other variables—high blood pressure, coronary heart disease, angina (or angina pectoris), myocardial infarction, and any other heart disease or condition. This denoted whether the individual had ever been diagnosed with any of these conditions or diseases [30,31].

Using SAS on Demand for Academics (SAS institute Inc., Cary, NC, USA), the characteristics between the cancer diagnosis variable were compared using the chi-square test. The relationship between BMI and cholesterol combinations (independent variable) and cancer (dependent variable) was assessed by using unadjusted and adjusted logistic regression models. The NWNC group was considered the reference group. The adjusted model accounted for potential covariates—sex, age, race, ethnicity, education level, employment status, marital status, alcohol and smoking status, diabetes, and cardiovascular disease. Alpha level was set a priori at 0.05. MEPS contains cluster, strata, and weighting variables which were used to maintain the structure of the complex survey data and to produce national estimates for the non-institutionalized US population [30].

## 3. Results

This study included a total of 20,818 individuals who met the eligibility criteria described above. Within this group, 2668 individuals were diagnosed with cancer, while other 18,150 were not. These two populations were weighted to correspond to 29,770,359 and 222,570,256 people in the US, respectively.

The characteristics of the study sample are presented in Table 1. There was a statistically significant difference between people who were diagnosed with cancer and the people who were not for all groups (*p* < 0.05). The most common independent groups were NWNC and OWNC followed by ONC and OHC. The UWHC and UWNC groups contained the fewest people in this study. There were approximately equal proportions of males and females. The majority of individuals were older than 30 years, white race, non-Hispanic ethnicity, with at least completed high school, married, unemployed, non-smokers, without diabetes, but with some kind of cardiovascular disease.

The findings from the logistic regression analyses are presented in Table 2. In the unadjusted logistic regression model, the OHC, OWHC, UWHC, and NWHC groups were each associated with higher odds of having a diagnosis of cancer than the NWNC (reference) group. The ONC group was associated with lower odds of having a diagnosis of cancer than the NWNC (reference) group. The unadjusted model had a Wald value of *p* < 0.0001 and a c-statistic of 0.616.

In the adjusted logistic regression model, only the UWHC and NWHC groups were associated with higher odds of having a diagnosis of cancer than the NWNC (reference) group. The ONC group remained associated with lower odds of having a diagnosis of cancer than the NWNC (reference) group. There was no statistical association for the remaining groups. The adjusted model had a Wald value of *p* < 0.0001 and a c-statistic of 0.765.

## 4. Discussion

The key findings from this study were that the UWHC group and the NWHC group were associated with higher odds of cancer, the ONC group were associated with lower odds of cancer, and there was no association between the remaining groups and cancer. Previously conducted studies often employed a prospective cohort design and focused on assessing the relationship between site-specific cancer and cholesterol, or site-specific cancer and BMI status [34,35,36,37]. Our retrospective, cross-sectional study focusses on the relationship of any cancer (i.e., not site-specific cancer) with a combination of cholesterol and BMI status using a nationally representative dataset (i.e., MEPS). This approach was intended to provide new insights into the association of cancer with cholesterol and BMI status that has not been fully assessed or properly understood. The comparison of our results requires careful consideration of not just the results given by previous authors but also of datasets and methodologies used. A discussion of our findings, and reasons to potentially explain them, follows.

Being overweight or underweight is a known risk factor for a shifted metabolism which may contribute to the development of severe pathologies [8]. It has previously been reported that increased BMI is an important predictor of cancer risk [26]. According to research from the American Cancer Society, excess body weight is thought to be responsible for about 11% of cancers in women and about 5% of cancers in men in the US [5]. In addition, excess body weight is attributable to about 7% of all cancer deaths [27]. Aside from cancer specifically, there is evidence to suggest that being underweight increases mortality and reduces life expectancy [38]. Therefore, we expected our results to show a positive association between overweight/obesity and having a cancer diagnosis. However, we found that the UWHC and NWHC groups were associated with higher odds of having cancer diagnosis compared to the NWNC group. There is some evidence that supports our findings in the literature. For instance, one small retrospective case–control study of 41 Indian women observed that the underweight population (based on BMI) had a higher risk of breast cancer development [39]. Furthermore, there is evidence that weight loss can help reduce the risk of cancer. For example, data from the Nurses’ Health Study demonstrated that women who lose weight after the menopause are at lower risk for breast cancer than women who maintain their weight [40]. In our study, we also found the ONC group was associated with lower odds of having a cancer diagnosis compared to the NWNC group. This is an unexpected finding given that existing evidence indicates a higher risk of cancer among people who are obese [8]. Additional studies have typically supported the intuitive claim that the risk of cancer is increased with higher BMI. For instance, a BMI in the range of 15–25 kg/m^2^ showed a negative correlation with lung and upper aerodigestive cancer, while a BMI in the range of 25–50 kg/m^2^ showed a positive correlation with other specified cancers [41]. Mili et al. [42] found that increased BMI was also associated with a multitude of site-specific cancers, and metabolic syndrome has been consistently and positively associated with the risk of common cancers such as colorectal, endometrial, and postmenopausal breast cancer. In contrast with these findings, data from over 100,000 individuals from the Danish general population found that high BMI was associated with a lower risk of lung and skin cancer overall yet a higher risk of breast cancer in postmenopausal women, though it had no association with other types of cancer [43]. Bjørge et al. [35] found that overweight patients had increased levels of obesity related cancers and that the risk of cancer was correlated with degree, timing, and duration of overweight and obesity.

A possible interpretation of our non-intuitive results may be that the relationship between weight and cancer is too complex to be described by a single underlying factor, such as BMI. That is, BMI itself does not uniquely describe the overall condition of a patient. Standardized “normal” BMI might be a misleading indicator in some cases. For instance, BMI does not distinguish between muscularity and body fat. By way of example, athletes with a high muscle mass may be considered overweight but not unhealthy. In these cases, waist circumference may be a more appropriate indicator to use [10,44]. To better answer our study question, future research could utilize alternative measures such as waist circumference alongside BMI to obtain a more holistic assessment of these characteristics and their association with cancer diagnosis. It is worth emphasizing that many studies, including ours, used self-reported BMI data. It is possible that people inaccurately reported their true height and weight, which in turn produced a biased estimate of their true BMI [45]. For instance, those who overestimated their height and underestimated their weight, would report a lower BMI than reality, which may have influenced our results.

Another consideration is the lack of a temporal component in our study and others [41]. It is possible that a lower body weight, or a decrease in body weight, does not lead to cancer, but rather is an effect of cancer or cancer treatment. We were also unable to capture in this study events that may have altered a person’s BMI. For example, weight loss through surgical reduction among obese patients is associated with reduced risks of cancer [46]. Furthermore, it may be that the use of any cancer in our study was too broad. It may be more insightful to assess the association of specific cancers with cholesterol and BMI status. This scenario is commonly encountered in various studies, such as the observed high BMI being associated with lower risk of lung and skin cancer overall and with higher risk of breast cancer in postmenopausal women, but not with other types of cancer [43]. A study of more than five million adults from the United Kingdom [47] found different effects for different types of cancer—being underweight was associated with increased risk of oral cavity, esophageal, stomach, and lung cancers, but a decreased risk of postmenopausal breast and prostate cancers.

With regard to cholesterol levels, further research is required to explain the association with cancer as there are contradictory data in the literature. Some studies support the claim that there is a positive relationship between increased cholesterol levels and a risk for certain cancer types [18,19,20], while others show no association between cancer and cholesterol [21,22,23,48]. Controversially, some studies have suggested a positive correlation between low cholesterol levels, and statins were speculated to have carcinogenic properties [24,25,49]. However, Lavigne et al. [50] assessed individuals who had not used lipid-lowering drugs and found that low levels of low-density lipoprotein (LDL) cholesterol can predate cancer diagnosis by decades and also suggested that there is no evidence to indicate that lowering cholesterol with a medication predisposes a person to cancer. Islam et al. [51] also stated there is no evidence that statin usage is linked to breast cancer, while Law et al. [52] suggested that dietary recommendations to lower serum cholesterol should be given unreservedly. Our results, that under- and normal-weighted people with increased cholesterol levels were associated with higher odds of having a cancer diagnosis and that obese people with normal cholesterol levels were associated with lower odds of having a cancer diagnosis, suggest that increased cholesterol level might be associated with cancer. However, the American Institute for Cancer Research [53] suggests that dietary cholesterol might not have direct effect on cancer risk, but rather food that is rich in cholesterol (red and processed meat, milk, cheese, and other dairy products) could influence cancer risk. Hence, it is not possible to exclude the possibility that increased cancer rates are not related directly to cholesterol levels, but rather a combination of more subtle influences that correlate with cholesterol. Many of the studies, as noted earlier, are not in agreement with our findings, implying instead that cholesterol levels are inversely associated with cancer meaning that people with low cholesterol levels are associated with higher risk of cancer diagnosis [17,36,54]. Yet, one of the older studies [55] suggests that the increased occurrence of cancer at low cholesterol levels might be due to the presence of preclinical cancer. Therefore, the findings from our study add to the contradictory existing literature and highlight the need for further investigation. Untangling different background influences that correlate with cholesterol might be the key to resolving apparent dissention in the literature.

This study had a large sample size that was successfully weighted to produce national estimates, an approach that enhances the representativeness and generalizability of our study findings. The limitations of the study are rooted in self-reported and secondary data analysis which can lead to bias, although frequent MEPS interviews (occurring approximately every five months) help limit these risks. Accounting for the aforementioned point that BMI is not always the best tool to assess obesity, this study has the potential issues of inaccurate self-reporting of either height, weight, or both, which could have influenced our results. Aside from internal biases, we cannot exclude the objective caveats. For example, the presence of familiar hypercholesterolemia might affect the results. Also, the dataset does not report on the laboratory diagnostic techniques used to measure cholesterol, leaving no quantifiable error estimates. On a separate note, the lack of a causal component makes it impossible to establish whether a cancer diagnosis predates the current BMI/cholesterol status or vice versa (i.e., the study design was not capable of establishing a cause–effect relationship but rather was capable of detecting a statistical association between BMI/cholesterol status and cancer status).

More data are needed on less-common types of cancer, alongside the need to investigate specific patient cohorts stratified by age so that obesity and cholesterol levels can be studied in relation to lifetime risk for specific cancers. It is also important to consider that obesity is not only marked by BMI but also other parameters, such as the distribution of body fat or waist circumference [56]. Research is also warranted with an additional dataset to investigate the effect of medications, particularly cholesterol-lowering medications (e.g., Statins), on the relationship of BMI and cholesterol status with cancer diagnosis. The limitations of our results with respect to the lack of a temporal component also raise the necessity of conducting a study with a prospective design that would also assess the relationship of BMI and cholesterol status with cancer diagnosis status. Although our study is highly specific in terms of combining BMI and cholesterol with respect to cancer diagnosis, our findings point to a large number of unexplored topics and motivations for future research in this field.

## 5. Conclusions

This study provides a unique set of data about the association between cancer and a combination of Body Mass Index and cholesterol level. Underweighted and normal weighted people with high cholesterol levels were found to be associated with higher odds of having cancer diagnosis, while obese people with normal cholesterol level were found to be associated with lower odds of having cancer diagnosis. To further explore and better understand this topic, we suggest more research with prospective design that includes temporal component, as well as considering site-specific cancers instead of considering all cancers combined.

## Figures and Tables

**Table 1:** Characteristics of study subjects stratified by cancer diagnosis status.

Factors	Cancer Diagnosed—Percent (95% Confidence Interval)	Cancer Not Diagnosed—Percent (95% Confidence Interval)	*p* Value
Independent			
Obese; High Cholesterol	20.0 (17.9, 22.0)	13.9 (13.1, 14.7)	<0.0001
Overweight; High Cholesterol	19.9 (17.9, 21.9)	11.1 (10.3, 11.8)	
Underweight; High Cholesterol	1.1 (0.5, 1.6)	0.4 (0.2, 0.5)	
Normal weight; High Cholesterol	14.9 (13.0, 16.8)	7.3 (6.7, 8.0)	
Obese; Normal Cholesterol	11.4 (9.8, 13.0)	20.3 (19.1, 21.4)	
Overweight; Normal Cholesterol	14.2 (12.3, 16.0)	21.4 (20.4, 22.4)	
Underweight; Normal Cholesterol	1.2 (0.7, 1.8)	1.5 (1.2, 1.8)	
Normal weight; Normal Cholesterol	17.3 (15.4, 19.2)	24.1 (22.9, 25.4)	
Gender			
Male	42.7 (40.4, 44.9)	49.1 (48.5, 49.8)	<0.0001
Female	57.3 (55.1, 59.6)	50.9 (50.2, 51.5)	
Age			
<30	2.0 (1.3, 2.6)	22.7 (21.8, 23.7)	<0.0001
≥30	98.0 (97.4, 98.7)	77.3 (76.3, 78.2)	
Race			
White	89.6 (88.2, 91.1)	76.1 (74.4, 77.8)	<0.0001
Black	6.6 (5.5, 7.8)	13.2 (12.0, 14.4)	
Asian/Hawaiian/Pacific Islands	2.1 (1.4, 2.8)	7.1 (6.0, 8.2)	
Other	1.6 (1.1, 2.1)	3.6 (3.1, 4.1)	
Ethnicity			
Hispanic	6.8 (5.6, 8.1)	18.2 (16.3, 20.1)	<0.0001
Not Hispanic	93.2 (91.9, 94.4)	81.8 (79.9, 83.7)	
Education			
Up to 12 years	33.1 (30.8, 35.5)	40.5 (39.0, 42.1)	<0.0001
Over 12 years	66.9 (64.5, 69.2)	59.5 (57.9, 61.0)	
Employment Status			
Employed	41.0 (38.6, 43.3)	69.2 (68.1, 70.2)	<0.0001
Unemployed	59.0 (56.7, 61.4)	30.8 (29.8, 31.9)	
Marital Status			
Married	59.0 (56.8, 61.3)	50.6 (49.5, 51.8)	<0.0001
Other	41.0 (38.7, 43.2)	49.4 (48.2, 50.5)	
Alcohol Consumption			
None	37.1 (34.1, 40.1)	37.6 (36.0, 39.2)	<0.0001
Up to 1 drink/week	36.6 (33.6, 39.6)	42.2 (41.1, 43.4)	
Over 1 drink/week	26.3 (23.6, 29.0)	20.1 (18.9, 21.3)	
Smoking Status			
Yes	10.3 (9.0, 11.7)	12.6 (11.8, 13.3)	0.0057
No	89.7 (88.3, 91.0)	87.4 (86.7, 88.2)	
Diabetes			
Yes	19.4 (17.6, 21.2)	10.6 (10.0, 11.1)	<0.0001
No	80.6 (78.8, 82.4)	89.4 (88.9, 90.0)	
Cardiovascular Disease			
Yes	64.6 (62.2, 67.1)	34.3 (33.4, 35.3)	<0.0001
No	35.4 (32.9, 37.8)	65.7 (64.7, 66.6)	

**Table 2:** Unadjusted and adjusted logistic regression results.

Factors	Unadjusted Odds Ratio (95% Confidence Interval)	Adjusted Odds Ratio (95% Confidence Interval)
Obese; High Cholesterol vs. Ref.	1.999 (1.676–2.384)	0.917 (0.734–1.145)
Overweight; High Cholesterol vs. Ref.	2.506 (2.107–2.980)	1.159 (0.933–1.440)
Underweight; High Cholesterol vs. Ref.	4.043 (2.243–7.290)	2.002 (1.032–3.885)
Normal weight; High Cholesterol vs. Ref.	2.834 (2.310–3.477)	1.326 (1.047–1.681)
Obese; Normal Cholesterol vs. Ref.	0.787 (0.647–0.957)	0.681 (0.543–0.853)
Overweight; Normal Cholesterol vs. Ref.	0.922 (0.757–1.122)	0.862 (0.691–1.074)
Underweight; Normal Cholesterol vs. Ref.	1.101 (0.637–1.902)	1.052 (0.541–2.045)

Ref. = normal weight or normal cholesterol. The values are adjusted for gender, age, race, ethnicity, education, employment status, marital status, alcohol consumption, smoking status, diabetes, and cardiovascular disease.

## Data Availability

Data are available from the corresponding author upon reasonable request.
